# Circulating fibroblast activation protein activity and antigen levels correlate strongly when measured in liver disease and coronary heart disease

**DOI:** 10.1371/journal.pone.0178987

**Published:** 2017-06-05

**Authors:** Shirley Uitte de Willige, Fiona M. Keane, David G. Bowen, Joyce J. M. C. Malfliet, H. Emma Zhang, Bharvi Maneck, Geoffrey W. McCaughan, Frank W. G. Leebeek, Dingeman C. Rijken, Mark D. Gorrell

**Affiliations:** 1 Department of Hematology, Erasmus University Medical Center, Rotterdam, The Netherlands; 2 Department of Molecular Hepatology, Centenary Institute, Sydney Medical School, University of Sydney, Sydney, New South Wales, Australia; 3 AW Morrow Gastroenterology and Liver Centre, Royal Prince Alfred Hospital, Sydney, New South Wales, Australia; Rosalind Franklin University of Medicine and Science, UNITED STATES

## Abstract

**Background and aim:**

Circulating fibroblast activation protein (cFAP) is a constitutively active enzyme expressed by activated fibroblasts that has both dipeptidyl peptidase and endopeptidase activities. We aimed to assess the correlation between cFAP activity and antigen levels and to compare variations in levels.

**Methods:**

In plasma of 465 control individuals, 368 patients with coronary heart disease (CHD) and 102 hepatitis C virus (HCV) infected patients with severe liver disease before and after liver transplant, cFAP activity levels were measured with a newly developed cFAP activity assay. In the same samples, cFAP antigen levels were measured using a commercially available cFAP ELISA. Correlation analyses between activity and antigen levels were performed by calculating Pearson’s correlation coefficient (ρ). Additionally, normal ranges, determinants and differences between cohorts and between anticoagulants were investigated.

**Results:**

cFAP activity and antigen levels significantly correlated in controls (ρ: 0.660, p<0.001) and in CHD patients (ρ: 0.709, p<0.001). cFAP activity and antigen levels in the HCV cohort were significantly lower in the samples taken after liver transplantation (p<0.001) and normalized toward levels of healthy individuals. Furthermore, cFAP activity and antigen levels were higher in men and significantly associated with body mass index. Also, cFAP activity and antigen levels were higher in EDTA plasma as compared to the levels in citrated plasma from the same healthy individuals.

**Conclusions:**

For analyzing cFAP levels, either activity levels or antigen levels can be measured to investigate differences between individuals. However, it is of importance that blood samples are collected in the same anticoagulant.

## Introduction

The serine protease fibroblast activation protein (FAP) is a member of the dipeptidyl peptidase (DPP) 4 family [1]. FAP is a constitutively active enzyme that has both DPP and endopeptidase activities and is expressed in tissues by activated fibroblasts. A derivative of FAP circulates in the blood (cFAP). Substrates of FAP include collagen type I [2, 3], fibroblast growth factor-21 [4], alpha-2-antiplasmin (α2AP) [5] and the neuropeptides B-type natriuretic peptide, neuropeptide Y, substance P and peptide YY [6, 7].

We recently developed a robust and sensitive assay to quantify cFAP activity in organs and fluids using the FAP-specific substrate 3144-amino methyl-coumarin (AMC) [8]. We showed that FAP is a highly promising biomarker, as both tissue and circulating levels are low in healthy individuals, but FAP is elevated in diseased organs and cFAP is elevated in cirrhosis [8, 9]. Similarly, elevated cFAP was restricted to subjects with moderate to severe fibrosis in patients with type 2 diabetes or undergoing bariatric surgery [9, 10]. Significantly, low cFAP values had a negative predictive value for grade ≥F2 fibrosis of 95% and cFAP activity measurement was used to re-classify more than 40% patients who had been classified as ‘indeterminate risk’ by the non-alcoholic fatty liver disease fibrosis score algorithm as ‘low risk of fibrosis’ [10]. These data indicate that cFAP can have clinical utility for increasing the precision of assessing which patients are at risk of fibrosis and thus require transient elastography. To further establish cFAP as a diagnostic biomarker for current and perhaps regressing liver fibrosis more investigation is needed.

In two other studies, we used a commercially available cFAP ELISA and showed increased cFAP antigen levels in liver disease [11] and equal cFAP antigen levels in patients with a recent arterial thrombotic event, including acute myocardial infarction and ischemic stroke [12]. The present study aimed to assess the correlation between our new cFAP activity assay that specifically measures cFAP enzyme activity and the commercial cFAP antigen assay that is specific for cFAP protein levels. We additionally investigated determinants of cFAP activity in healthy control individuals and examined differences in cFAP levels between individuals. For this we used the plasma samples of 3 different cohorts: 1] a control cohort consisting of healthy individuals, 2] a cohort of patients with coronary heart disease (CHD), containing patients with acute myocardial infarction or unstable angina pectoris, and 3] a cohort of hepatitis C virus (HCV) infected patients with severe liver disease of whom blood was taken before and/or after a liver transplant. The results show a strong correlation between the activity levels and the antigen levels of cFAP.

## Materials and methods

### Plasma samples

The control cohort and the CHD cohort derived from the ‘Genetic risk factors for Arterial Thrombosis at young age: the role of TAFI and other Coagulation factors’ (ATTAC) study, which has been described previously [13]. In short, 391 consecutive patients who presented with a first arterial thrombotic event at young age (males: 45 years or younger, females: 55 years or younger) were included. Control subjects (n = 465) had no history of cardiovascular disease. Blood samples were collected in both 0.1 volume of sodium citrate (0.105 mol/L) and in EDTA (final concentration: 6.16 mmol/L). The HCV cohort samples consisted of 142 plasma samples anticoagulated with EDTA (final concentration: 6.16 mmol/L) from 93 patients with Child-Pugh C cirrhosis pre-transplant (S1 Table). From 33 HCV patients, two or more samples were obtained at different time points. For direct comparison, both citrate and EDTA anticoagulated blood samples were additionally collected from 21 Australian healthy individuals. All subjects provided written informed consent prior to undertaking any study procedures. All procedures were approved by the Medical Ethics Committee of Erasmus MC and the Sydney Local Health District Human Research Ethics Committee and were in accordance with the Helsinki Declaration.

### cFAP activity assay

Circulating FAP activity levels were measured using a recently developed in-house enzyme-activity assay as described and validated [8]. Fluorescent standards in a linear range of 0–600 pmol AMC were used to interpolate cFAP activity in a 100-fold diluted plasma sample using the FAP-specific substrate 3144-AMC at 150 μM. Inter- and intra-assay variation was 10.7% and 4.5%, respectively. The activity of cFAP was converted to ng mL^-1^ with a standard curve of recombinant human FAP (R&D Systems, Abingdon, UK) ranging from 0–400 ng mL^-1^ diluted in PBS.

### cFAP antigen assay

Circulating FAP antigen levels were measured using the human FAP DuoSet^®^ ELISA Development kit (R&D Systems, Abingdon, UK), according to the manufacturer’s protocol, as described [12, 14]. The cFAP level of a 200-fold diluted plasma sample was measured in duplicate and was calculated as the mean result of the two measurements. The assay was calibrated using recombinant human FAP (R&D Systems, Abingdon, UK) as standard. Inter- and intra-assay variation was 12.5% and 3.5%, respectively.

### Statistical analyses

Distributions of the cFAP activity and cFAP antigen levels in the cohorts were tested for normality using the Kolmogorov-Smirnov test. Natural log transformation was required to obtain normal distributions. Data are presented as geometric mean and geometric standard deviation (SD). Significance of correlation between cFAP activity and antigen levels were investigated using Pearson’s correlation coefficient (ρ). In the control cohort, normal ranges for cFAP activity and antigen levels were calculated as geometric mean ± 2 geometric SD (i.e. 95% confidence interval). Differences in cFAP levels between cohorts or between different anticoagulants (citrate and EDTA) were tested for significance using (paired) Student’s t-test on the log-transformed data. Adjustments for confounders were made using linear regression. P-values <0.05 were considered statistically significant. All statistical analyses were carried out using SPSS software version 21.

## Results

### Sample characteristics

The baseline characteristics of the control individuals and CHD cohort have been published previously [12]. In general, the control individuals were slightly younger (39 years, range: 18–57) than the CHD patients (44 years, range: 23–55). The control cohort contained 63.5% females while the CHD cohort contained 44.0% females. The individuals of the HCV cohort were older (57 years, range: 31–77) and predominantly male (15.8% females).

### Correlation analyses

**Fig 1:**
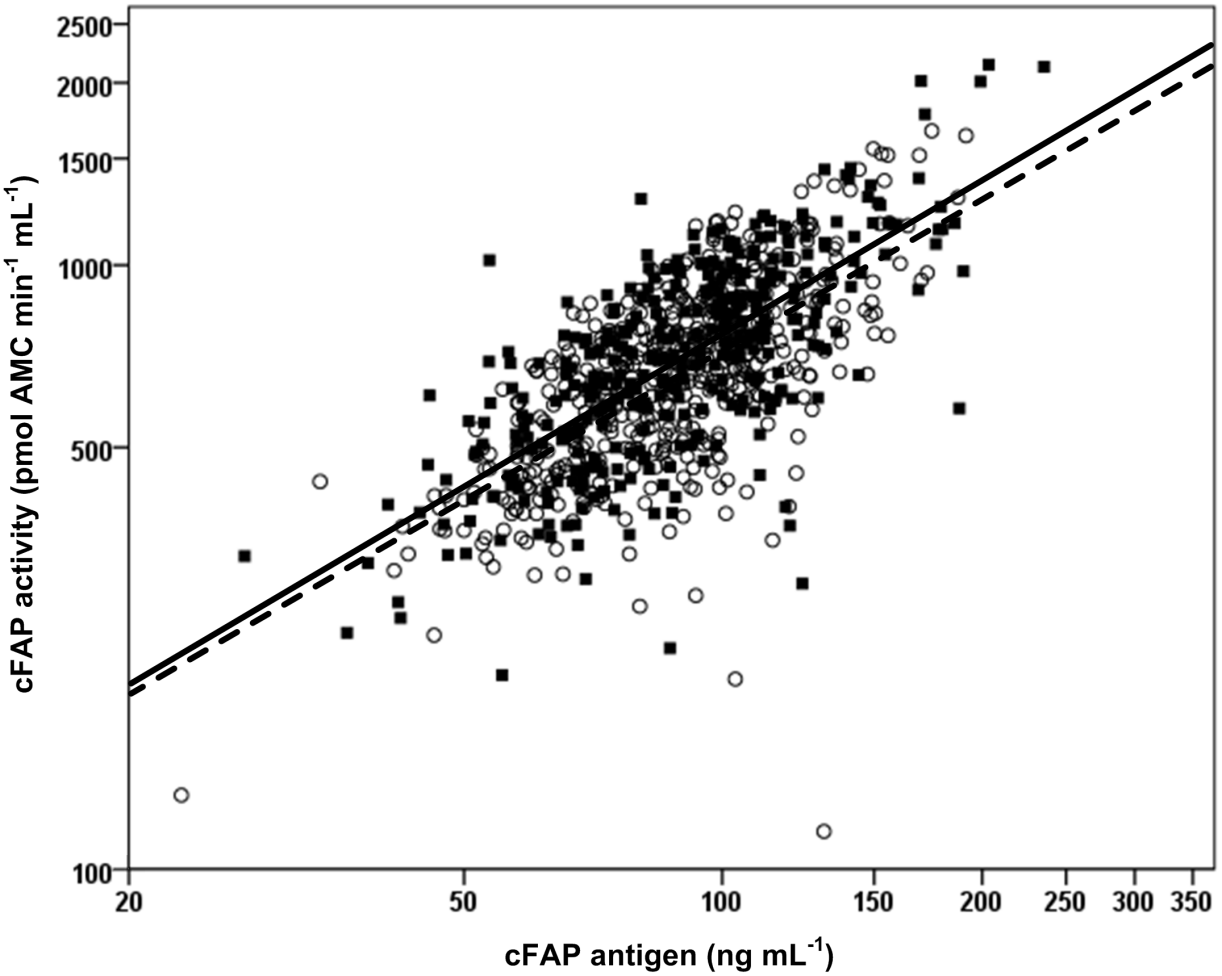
Correlation between sFAP activity levels and cFAP antigen levels. Control cohort (n = 465): open circles and dashed line (r^2^ = 0.435). CHD cohort (n = 368): closed squares and solid line (r^2^ = 0.503). The axes are on a log scale.

The scatterplot (Fig 1) illustrates the significant correlation between cFAP activity and antigen levels. Similar correlation coefficients were found for the separate cohorts; ρ: 0.660, p<0.001 in the control cohort and ρ: 0.709, p<0.001 in the CHD cohort, indicating that the correlation between the assays is not influenced by CHD.

To compare cFAP antigen and activity levels in similar units, cFAP activity levels were converted from pmol AMC min^-1^ mL^-1^ to ng mL^-1^. On average, cFAP antigen levels were 4.7-fold higher than the cFAP activity levels in ng mL^-1^.

### Normal ranges

As we have measured both cFAP activity and cFAP antigen levels in a large cohort of 465 healthy control individuals, we calculated normal ranges based on the 95% confidence interval of the values in this cohort. We defined the normal range of cFAP activity to be 316–1360 pmol AMC/min/ml. The cFAP antigen normal range was defined as 49–159 ng/ml.

### Determinants of cFAP activity level

In the control cohort we additionally investigated determinants of cFAP activity, as previously performed for cFAP antigen levels [12]. As expected due to the strong correlation between the assays, the determinants of cFAP activity were similar to the determinants of cFAP antigen levels. Men had significantly higher cFAP activity levels than women (Table 1). Furthermore, there was a significant positive association between body mass index (BMI) and cFAP activity level, Spearman’s rho: 0.168, p<0.001.

**Table 1:** cFAP activity and antigen levels in the different cohorts.

Cohort	cFAP activity (pmol AMC min^-1^ mL^-1^)	P-value^†^	cFAP antigen (ng mL^-1^)	P-value^‡^
Male controls^§^ (n = 166)	704 ± 273	<0.001	95 ± 29	<0.001[12]
Female controls^§^ (n = 299)	627 ± 224		84 ± 24	
Control cohort^§^ (n = 465)	656 ± 244	0.561	88 ± 26	0.849[12]
CHD cohort^§^ (n = 368)	696 ± 272		89 ± 29	
HCV cohort^¶^ (pre-Tx, n = 32)	1625 ± 613	<0.001	146 *±* 49	<0.001
HCV cohort^¶^ (post-Tx, n = 110)	1055 ± 463		103 ± 45	
EDTA plasma (n = 21^†^; n = 25^‡^)	1087 ± 287	<0.001	129 ± 42	0.001
Citrate plasma (n = 21^†^; n = 25^‡^)	893 ± 214		113 ± 43	

P-values are adjusted for age and gender. cFAP: circulating fibroblast activation protein; Tx: transplant;

^†^cFAP activity;

^‡^cFAP antigen;

^§^citrated plasma;

^¶^EDTA plasma.

### Differences in cFAP activity levels between cohorts

We compared the levels between the different cohorts and found that the cFAP activity levels in the control cohort were similar to those in the CHD cohort, p = 0.561 after adjustment for age and gender (Table 1). Additional adjustment for BMI did not change this, p = 0.800. These results are in line with our previous study in which we showed no differences in cFAP antigen levels between healthy individuals and CHD patients [12].

In the HCV cohort (S1 Table) we compared samples taken before liver transplantation (n = 32) with samples taken after liver transplantation (n = 110). To allow clearance of the pre-transplant cFAP and for immunosuppressive treatment to stabilize, samples taken up to 6 months after liver transplantation were excluded. Both cFAP activity levels and cFAP antigen levels were significantly lower in the samples taken after liver transplantation, p<0.001 after adjustment for age and gender (Table 1) and normalized toward the levels found in the plasma of control individuals.

### Differences in cFAP levels between anticoagulants

cFAP levels of the control and CHD cohorts were measured in citrated plasma, whereas the cFAP levels of the HCV cohort were measured in EDTA plasma. Therefore, we additionally tested whether cFAP levels differed between the two anticoagulants. We found that cFAP levels, activity as well as antigen, were significantly greater in EDTA plasma compared to citrated plasma (Table 1).

## Discussion

In this study we showed that cFAP antigen levels (detected by ELISA) correlate strongly with cFAP enzyme activity (detected by synthetic fluorescence substrate assay). This suggests that the proportion of cFAP protein measured by this ELISA is enzymatically active and stable. The data imply that there are no endogenous inhibitors of cFAP and that cFAP stability is not affected by major influences such as the presence of cirrhosis or immunosuppression.

Conversion of the activity data into ng mL^-1^ showed that on average the antigen levels were 4.7-fold higher than the activity levels. This may be explained by the fact that only fully intact cFAP homodimers have activity [15], whereas the ELISA will measure various forms of cFAP. In plasma some of the FAP will be monomeric or fragments that can be detected by the ELISA but will not be measured by our activity assay. However, we showed that both assays are reliable for detecting differences between individuals and cohorts. The data presented in this study is pertinent only to the specific antibodies used in the ELISA employed here. Other antibodies may give different results.

We previously showed that, with adjustment for age and gender, cFAP is not significantly different in CHD patients and in control individuals [12], and this finding is now independently confirmed using the FAP activity assay. In addition, we recently showed that neither rheumatoid arthritis nor systemic sclerosis alter cFAP activity levels [9]. Interestingly, the present study showed for the first time that cFAP levels decrease following the establishment of a healthy transplanted donor liver that replaced a cirrhotic liver. Our recent data indicates that lacking FAP does not alter immunity to influenza in mice [16]. However, FAP-expressing cells have been associated with impaired immunity in mice [17], so cFAP might potentially be influenced by the immunosuppression that is administered post-transplant. Therefore, we examined cFAP only 6 months or more post-transplant. As there were only three individuals from whom we had a plasma sample obtained both before and after liver transplantation, we could not perform paired analyses. Nevertheless, the observation that after liver transplantation cFAP levels normalized toward levels of healthy individuals supports growing evidence that activated stellate cells and activated myofibroblasts in the liver, which are prevalent in cirrhosis, are major sources of cFAP above the levels found in healthy individuals [18, 19]. Our previous work showed that cFAP in liver cirrhosis patients is elevated, with highest levels when fibrosis is worst (Child-Pugh C) [8, 11], and that low cFAP has a 95% negative predictive value for ≥F2 fibrosis [10]. Thus, cFAP may have clinical utility as a biomarker for progression and regression of liver fibrosis.

The cFAP activity levels positively associated with male gender and with BMI. Both observations might relate to hormonal influence, and an analysis of the FAP gene promotor supports this concept [20]. FAP can inactivate the starvation hormone FGF21 in humans, and FAP deficient mice and FAP-inhibited mice are leaner than controls (Gorrell MD, unpublished results) [21], so elevated cFAP might associate with BMI due to a metabolic function of FAP such as FGF21 inactivation.

Furthermore, we found higher cFAP activity and antigen levels in EDTA plasma as compared to the levels in citrated plasma from the same healthy individuals, suggesting that the anticoagulant may interfere with the cFAP level. However, as the 0.1 volume of citrate can dilute the plasma up to 18% (assuming an average hematocrit of 45%) this may explain the higher values found in undiluted EDTA plasma. Accordingly, the cFAP activity levels in undiluted EDTA plasma and serum do not differ [8]. The results of our studies are not influenced by this dilution, as the same anticoagulants were used within the cohorts. Furthermore, the levels of the patients of the pre-transplant HCV cohort are higher than can be explained by the anticoagulant dilution only.

In summary, we show that for analyzing cFAP levels, either activity levels or antigen levels can be measured and that it is of importance that blood samples are collected in the same anticoagulant.

## Supporting information

S1 Data Main database. (XLSX)

S2 Data Database EDTA and citrate levels. (XLSX)

S1 Table General characteristics of the liver transplant clinic hepatitis C patients. Pt: patient; Tx: transplant; NG: Not given on patient record; ND: not detected.
(PDF)
