# Supplementary material for: Circulating fibroblast activation protein activity and antigen levels correlate strongly when measured in liver disease and coronary heart disease
Source: PLoS One. 2017 Jun 5;12(6):e0178987. doi: 10.1371/journal.pone.0178987 (PMC5459491; doi:10.1371/journal.pone.0178987)
Supplement: S1 Table — Pt: patient; Tx: transplant; NG: Not given on patient record; ND: not detected. (PDF) [file pone.0178987.s003.pdf]

S1 Table. General characteristics of the liver transplant clinic hepatitis C patients.

Pt No	Gender	Age at Sample	Pre/Post Tx	Viral Load (IU mL^{-1})	HCV Genotype	Bilirubin (µmol L^{-1})	Albumin (g L^{-1})	ALT (U L^{-1})	AST (U L^{-1})	Platelet (No. L^{-1})
1	M	58.6	Post-Tx	46,300	1a	12	44	88	62	138
1	M	59.4	Post-Tx	138,000	1a	12	43	85	59	134
1	M	59.6	Post-Tx	187,000	1a	12	44	88	62	138
2	M	56.7	Post-Tx	25,600	4e	49	22	50	132	63
3	M	56.2	Pre-Tx	NG	1a/1b	21	29	27	48	58
3	M	56.4	Pre-Tx	NG	1a/1b	29	30	96	140	59
4	M	57.8	Post-Tx	ND	3a	6	44	39	33	296
5	M	60.7	Post-Tx	90,500,000	1b	20	44	159	297	97
6	M	52.3	Pre-Tx	108,000	1a	80	26	88	167	72
6	M	52.4	Pre-Tx	73,200	1a	83	26	86	168	65
7	M	54.3	Pre-Tx	NG	NG	67	20	41	75	100
8	M	50.2	Post-Tx	ND	3a	7	48	10	18	219
9	M	63.1	Pre-Tx	621,000	3a	39	30	96	131	52
9	M	64.1	Pre-Tx	NG	3a	68	33	90	136	48
10	M	42.2	Pre-Tx	5,570	1b	25	32	18	27	90
10	M	42.5	Pre-Tx	<15	1b	15	38	10	27	118
11	M	53.1	Pre-Tx	831,000	1	47	26	58	108	37
12	M	53.6	Post-Tx	926,000	3a	13	37	179	114	98
13	M	54.6	Post-Tx	217,000	3a	7	46	42	32	140
14	M	45.9	Pre-Tx	1,320,000	3a	29	38	71	111	23
16	M	59.9	Pre-Tx	NG	NG	6	46	27	22	245

16	M	60.0	Pre-Tx	NG	NG	3	43	40	25	554
16	M	60.9	Post-Tx	ND	NG	10	46	30	33	299
17	M	59.1	Post-Tx	4,060,000	3a	24	39	59	34	180
17	M	59.3	Post-Tx	4,120,000	3a	18	37	94	67	134
17	M	59.5	Post-Tx	711,000	3a	27	39	82	58	153
17	M	60.0	Post-Tx	6,560,000	3a	164	36	143	97	177
17	M	60.6	Post-Tx	5,940,000	3a	33	39	74	57	193
19	M	58.1	Post-Tx	NG	3a	10	48	26	24	145
19	M	58.9	Post-Tx	NG	3a	6	47	28	22	146
20	M	59.8	Pre-Tx	NG	3a	10	40	102	90	41
21	M	59.2	Post-Tx	6,350,000	1b	13	46	65	56	325
21	M	59.9	Post-Tx	5,150,000	1b	7	44	94	NG	293
21	M	61.1	Post-Tx	NG	1b	9	47	67	46	310
22	M	52.1	Pre-Tx	NG	3a	22	42	128	117	72
23	M	60.8	Pre-Tx	526,000	1	17	41	148	116	60
23	M	61.8	Pre-Tx	638,000	1	12	40	222	154	66
23	M	62.7	Pre-Tx	1,110,000	1	14	42	181	115	57
24	M	58.9	Post-Tx	302,000	NG	6	45	83	37	155
25	M	60.2	Pre-Tx	149,000	4c/4d	33	27	55	88	29
25	M	60.6	Pre-Tx	1,600,000	4c/4d	35	29	43	71	33
25	M	61.0	Pre-Tx	444,000	4c/4d	39	28	43	76	52
25	M	62.2	Post-Tx	630,000	4c/4d	8	41	26	20	84
26	M	63.9	Post-Tx	NG	1	6	43	42	42	221
26	M	64.2	Post-Tx	1,680,000	1	10	45	49	46	234
26	M	65.2	Post-Tx	NG	1	11	46	50	46	233

27	M	56.6	Post-Tx	NG	NG	14	43	33	23	131
28	M	48.7	Post-Tx	NG	1	7	47	56	54	264
29	M	54.4	Post-Tx	41900.00	1	NG	NG	NG	NG	NG
30	M	50.9	Post-Tx	7,560,000	3a	12	40	24	25	255
31	M	58.7	Post-Tx	2,550,000	2a/2c	25	40	76	94	58
32	F	72.2	Post-Tx	250,000	1a/1b	13	42	149	103	115
33	M	52.6	Post-Tx	1,300,000	1b	25	28	72	37	170
34	M	56.3	Post-Tx	4,070,000	1b	58	40	112	186	91
34	M	58.2	Post-Tx	660	1b	30	40	28	40	70
35	M	63.1	Post-Tx	3,160,000	3a	21	36	79	75	209
36	M	60.6	Post-Tx	NG	3a	7	44	29	36	154
37	M	53.5	Post-Tx	4,940,000	3a	10	48	219	107	146
37	M	53.6	Post-Tx	12,000,000	3a	9	43	203	100	154
38	M	44.3	Post-Tx	ND	3	10	46	69	47	76
38	M	44.3	Post-Tx	NG	3	14	47	109	57	91
38	M	44.4	Post-Tx	NG	3	13	43	98	57	94
38	M	45.7	Post-Tx	ND	3	15	45	33	23	111
39	M	53.0	Post-Tx	647,000	1a	9	51	91	42	170
40	M	64.6	Post-Tx	10,100	3a	9	47	45	28	173
41	M	59.1	Pre-Tx	NG	4h	14	33	79	87	89
42	F	57.0	Post-Tx	6,890,000	1	14	45	259	166	113
44	M	58.6	Pre-Tx	NG	1a/1b	21	30	97	134	86
44	M	58.9	Pre-Tx	NG	1a/1b	NG	NG	NG	NG	NG
45	F	50.3	Pre-Tx	NG	3a	NG	NG	NG	NG	NG
46	F	47.3	Post-Tx	NG	NG	4	39	10	12	187

47	M	68.7	Post-Tx	7,350,000	1b	29	33	121	147	93
47	M	70.4	Post-Tx	1,220,000	1b	36	30	63	62	43
48	F	67.5	Post-Tx	379,000	NG	11	39	54	37	100
48	F	68.1	Post-Tx	462,000	NG	10	38	64	31	107
49	M	52.1	Post-Tx	7,480	3a	12	39	50	52	85
50	M	47.9	Post-Tx	NG	3a	10	53	146	81	146
51	M	56.5	Post-Tx	960	3a	6	35	48	32	70
51	M	56.7	Post-Tx	NG	3a	5	33	53	34	38
52	F	61.8	Post-Tx	460,000	3b	13	37	61	94	152
53	F	76.6	Post-Tx	NG	6a	20	40	154	134	133
53	F	77.2	Post-Tx	777,000	6a	NG	NG	NG	NG	NG
54	M	60.2	Post-Tx	NG	3a	13	44	40	55	201
54	M	61.2	Post-Tx	5,260,000	3a	17	42	48	55	210
55	M	53.0	Post-Tx	ND	NG	3	47	27	25	141
56	M	58.8	Post-Tx	NG	3a	9	33	40	43	113
56	M	60.1	Post-Tx	NG	3a	9	31	39	48	66
57	M	53.8	Post-Tx	21,500,000	1	14	45	42	28	123
57	M	55.0	Post-Tx	6,570,000	1	10	46	91	58	121
57	M	55.2	Post-Tx	NG	1	NG	NG	NG	NG	NG
58	M	55.3	Post-Tx	NG	1a	10	42	22	20	138
58	M	55.6	Post-Tx	NG	1a	17	44	20	24	137
59	M	52.9	Post-Tx	35,600,000	1b	10	46	66	54	107
59	M	53.7	Post-Tx	3,520,000	1b	9	46	61	58	137
60	F	70.0	Post-Tx	811,000	4	5	40	68	75	135
61	M	57.5	Post-Tx	NG	1a	25	32	31	57	74

64	M	49.0	Pre-Tx	NG	1a	27	39	27	36	42
64	M	50.1	Post-Tx	NG	1a	7	43	28	24	50
65	M	51.3	Post-Tx	10,500,000	1a	22	43	148	41	91
66	F	55.1	Post-Tx	NG	NG	4	44	22	26	99
67	F	56.2	Post-Tx	6,830,000	3a	9	46	134	152	160
67	F	56.9	Post-Tx	16,500,000	3a	4	40	25	30	144
67	F	57.2	Post-Tx	NG	3a	5	40	21	30	133
68	M	60.1	Post-Tx	NG	1a	7	32	46	72	124
69	M	55.0	Pre-Tx	NG	1a/1b	NG	NG	NG	NG	NG
70	M	52.6	Pre-Tx	NG	1a	NG	NG	NG	NG	NG
71	M	49.3	Post-Tx	30,400,000	3a	5	41	130	97	121
72	M	52.1	Post-Tx	NG	1b	11	40	41	42	80
73	M	54.8	Post-Tx	NG	4h	10	45	35	35	89
74	M	51.3	Pre-Tx	NG	NG	64	28	81	133	67
75	M	66.7	Post-Tx	NG	NG	7	41	22	21	68
77	M	52.8	Pre-Tx	NG	NG	9	41	27	37	124
78	F	68.0	Post-Tx	NG	4c/4d	7	45	27	28	263
79	F	57.6	Post-Tx	6,140,000	3a	20	45	167	141	97
79	F	57.9	Post-Tx	7,760,000	3a	10	48	88	72	91
80	M	61.5	Post-Tx	14,000,000	1a/1b	10	44	484	351	119
80	M	62.6	Post-Tx	4,390,000	1a/1b	11	39	420	242	105
81	M	61.0	Post-Tx	ND	NG	9	47	17	21	150
82	F	54.8	Post-Tx	ND	NG	6	38	61	57	208
82	F	55.6	Post-Tx	ND	NG	NG	NG	NG	NG	NG
83	M	58.7	Post-Tx	NG	NG	17	41	48	67	164

84	M	31.2	Post-Tx	NG	NG	13	40	99	157	75
86	M	60.0	Post-Tx	NG	NG	15	47	219	138	150
86	M	61.9	Post-Tx	NG	NG	18	37	120	240	156
87	F	62.1	Post-Tx	NG	NG	24	39	28	31	20
88	M	58.8	Pre-Tx	NG	3a	28	27	111	140	NG
89	M	64.4	Post-Tx	NG	NG	42	37	187	220	50
90	M	45.9	Post-Tx	792,000	3a	5	46	91	36	264
91	M	58.1	Post-Tx	NG	1a	6	49	9	22	202
92	F	52.0	Pre-Tx	NG	1a/1b	18	30	142	165	NG
93	F	66.3	Post-Tx	2,480,000	4	6	51	46	31	143
93	F	66.9	Post-Tx	2,880,000	4	7	46	25	27	135
93	F	67.3	Post-Tx	NG	4	NG	NG	NG	NG	NG
94	M	76.5	Post-Tx	NG	1b	19	40	150	178	115
95	F	60.3	Post-Tx	NG	2b	9	45	30	34	98
96	M	64.8	Post-Tx	10,000,000	1b	9	50	27	30	202
99	F	46.3	Post-Tx	2,420,000	1	9	43	100	91	351
100	M	52.2	Post-Tx	2,870	3a	34	42	34	42	39
100	M	52.4	Post-Tx	450,000	3a	30	43	151	131	43
100	M	52.4	Post-Tx	325,000	3a	24	35	27	61	121
101	M	55.2	Post-Tx	NG	1	NG	NG	NG	NG	NG
102	M	69.9	Post-Tx	NG	1b	14	43	38	36	198

Pt: patient; Tx: transplant; NG: Not given on patient record; ND: not detected.
